# Phylogenomic analysis reveals five independently evolved African forage grass clades in the genus *Urochloa*

**DOI:** 10.1093/aob/mcae022

**Published:** 2024-02-14

**Authors:** Lizo E Masters, Paulina Tomaszewska, Trude Schwarzacher, Jan Hackel, Alexandre R Zuntini, Pat Heslop-Harrison, Maria S Vorontsova

**Affiliations:** Department of Genetics and Genome Biology, Institute for Environmental Futures, University of Leicester, Leicester LE17RH, UK; Accelerated Taxonomy/Trait Diversity and Function, Royal Botanic Gardens, Kew, Richmond, Surrey, TW9 3AB, UK; Department of Genetics and Genome Biology, Institute for Environmental Futures, University of Leicester, Leicester LE17RH, UK; Department of Genetics and Cell Physiology, University of Wroclaw, 50-328 Wroclaw, Poland; Department of Genetics and Genome Biology, Institute for Environmental Futures, University of Leicester, Leicester LE17RH, UK; Key Laboratory of Plant Resources Conservation and Sustainable Utilization/Guangdong Provincial Key Laboratory of Applied Botany, South China Botanical Garden, Chinese Academy of Sciences, Guangzhou, 510650, China; Accelerated Taxonomy/Trait Diversity and Function, Royal Botanic Gardens, Kew, Richmond, Surrey, TW9 3AB, UK; Department of Biology, University of Marburg, Karl-von-Frisch-Straße 8, 35043 Marburg, Germany; Accelerated Taxonomy/Trait Diversity and Function, Royal Botanic Gardens, Kew, Richmond, Surrey, TW9 3AB, UK; Department of Genetics and Genome Biology, Institute for Environmental Futures, University of Leicester, Leicester LE17RH, UK; Key Laboratory of Plant Resources Conservation and Sustainable Utilization/Guangdong Provincial Key Laboratory of Applied Botany, South China Botanical Garden, Chinese Academy of Sciences, Guangzhou, 510650, China; Accelerated Taxonomy/Trait Diversity and Function, Royal Botanic Gardens, Kew, Richmond, Surrey, TW9 3AB, UK

**Keywords:** *Urochloa*, *Brachiaria*, crop wild relatives, forage traits, phylogenomics, species tree, tropical forage systems

## Abstract

**Background and Aims:**

The grass genus *Urochloa* (*Brachiaria*) *sensu lato* includes forage crops that are important for beef and dairy industries in tropical and sub-tropical Africa, South America and Oceania/Australia. Economically important species include *U. brizantha*, *U. decumbens*, *U. humidicola*, *U. mutica*, *U. arrecta*, *U. trichopus*, *U. mosambicensis* and *Megathyrsus maximus*, all native to the African continent. Perennial growth habits, large, fast growing palatable leaves, intra- and interspecific morphological variability, apomictic reproductive systems and frequent polyploidy are widely shared within the genus. The combination of these traits probably favoured the selection for forage domestication and weediness, but trait emergence across *Urochloa* cannot be modelled, as a robust phylogenetic assessment of the genus has not been conducted. We aim to produce a phylogeny for *Urochloa* that includes all important forage species, and identify their closest wild relatives (crop wild relatives). Finally, we will use our phylogeny and available trait data to infer the ancestral states of important forage traits across *Urochloa s.l.* and model the evolution of forage syndromes across the genus.

**Methods:**

Using a target enrichment sequencing approach (Angiosperm 353), we inferred a species-level phylogeny for *Urochloa s.l.*, encompassing 54 species (~40 % of the genus) and outgroups. Phylogenies were inferred using a multispecies coalescent model and maximum likelihood method. We determined the phylogenetic placement of agriculturally important species and identified their closest wild relatives, or crop wild relatives, based on well-supported monophyly. Further, we mapped key traits associated with *Urochloa* forage crops to the species tree and estimated ancestral states for forage traits along branch lengths for continuous traits and at ancestral nodes in discrete traits.

**Key Results:**

Agricultural species belong to five independent clades, including *U. brizantha* and *U. decumbens* lying in a previously defined species complex. Crop wild relatives were identified for these clades supporting previous sub-generic groupings in *Urochloa* based on morphology. Using ancestral trait estimation models, we find that five morphological traits that correlate with forage potential (perennial growth habits, culm height, leaf size, a winged rachis and large seeds) independently evolved in forage clades.

**Conclusions:**

*Urochloa s.l.* is a highly diverse genus that contains numerous species with agricultural potential, including crop wild relatives that are currently underexploited. All forage species and their crop wild relatives naturally occur on the African continent and their conservation across their native distributions is essential. Genomic and phenotypic diversity in forage clade species and their wild relatives need to be better assessed both to develop conservation strategies and to exploit the diversity in the genus for improved sustainability in *Urochloa* cultivar production.

## INTRODUCTION

**Fig. 1.**
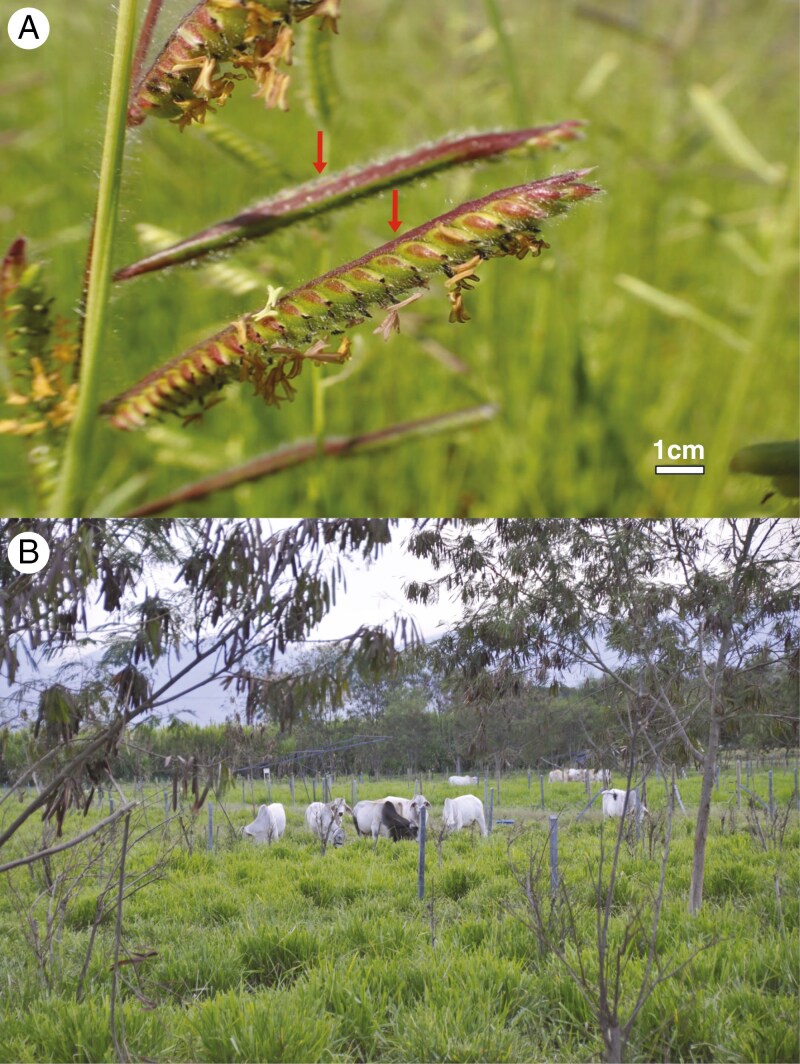
(A) Inflorescence with spikelets of *Urochloa decumbens*. The broadly winged rachises are indicated with red arrows. (B) Field with cultivated *Urochloa* sp. and grazing cattle in Cali, Colombia.

African grasses have been recognized for their forage potential since the 18th century, and as a result have been transplanted around the globe to upscale beef and dairy production for small-scale and commercial farms (Hartley and Williams, 1956; Parsons, 1972; Cook and Dias, 2006; Visser *et al.*, 2016). Today, arguably the most important of these African grasses belong to the genus *Urochloa* P. Beauv. (Family: Poaceae, Subfamily: Panicoidaea, Tribe: Paniceae, Subtribe: Melinidinae) (Kellogg, 2015; Soreng *et al.*, 2017) (Fig. 1). This large and diverse genus includes taxa previously placed in *Brachiaria* (Trin.) Briseb., *Chaetium* Nees, *Eriochloa* Kunth, *Scutachne* Hitchc. & Chase and *Megathyrsus* (Pilg.) B.K. Simon & S.W.L. Jacobs (Salariato *et al.*, 2010, 2012; Jank *et al.*, 2014; Kellogg, 2015; Namazzi *et al.*, 2020; Ferreira *et al.*, 2021). *Urochloa* forages are strongly preferred in sub-tropical and tropical regions as they are highly palatable and nutrient dense, are tolerant of low-quality soils, and outcompete alternative forage grasses in terms of biomass productivity such as *Pennisetum purpureum* Schumach. and *Cenchrus ciliaris* Fig. & De Not. (Maass *et al.*, 2015; Baptistella *et al.*, 2020; Njarui *et al.*, 2021; Rathore *et al.*, 2022). Since the 1950s these grasses have been adopted in forage systems in Southeast Asia, Australia, and especially Central and South America, with an estimated 99 million hectares of land devoted to *Urochloa* production in Brazil alone (ANUALPEC, 2008; Jank *et al.*, 2014).

Livestock rearing for meat and dairy are key areas of economic importance in numerous developing nations, contributing greatly to the livelihoods of rural communities (e.g. Colombia) and commercial farmers (e.g. in Brazil) (Jank *et al.*, 2014; Enciso *et al.*, 2021). Recent breeding projects led by the Centro Internacional de Agricultura Tropical (CIAT; now Alliance Biodiversity & CIAT), Colombia, and Empresa Brasileira de Pesquisa Agropecuária (EMBRAPA), Brazil, have produced highly productive and globally competitive *Urochloa* cultivars (do Valle and Savidan, 1996; Miles and do Valle, 1996; do Valle *et al*., 2013; Worthington and Miles, 2015; Espitia *et al*., 2020). High demand has resulted in the reintroduction of *Urochloa* cultivars into African countries including Kenya, Uganda and Madagascar, largely for improving milk production in small-holder farms (Maass *et al.*, 2015; Mutimura *et al.*, 2018).

Cattle rearing contributes greatly to global methane emissions, and the conversion of natural habitats to agricultural forage lands has contributed to global biodiversity loss (Herrero *et al.*, 2013; Peters *et al.*, 2013; Godfray *et al.*, 2018). African *Urochloa* forage species are often recognized as invasives in Australia and the Americas (Foxcroft *et al.*, 2010; Seabloom *et al.*, 2013; Visser *et al.*, 2016; Overholt and Franck, 2017). African *Urochloa* species aggressively exclude indigenous vegetation in disturbed environments through a combination of rapid growth and spread, and the production of allelopathic chemicals which hinder the development indigenous flora (Barbosa *et al.*, 2008; Kato-Noguchi *et al.*, 2014; Damasceno *et al.*, 2018). Further, *Urochloa* forages possess agriculturally undesirable traits, chiefly an inability to survive frost, and are typically grown in monocultures in large-scale systems (do Valle *et al.*, 2013; Krahl and Marocco, 2019). Although recent decades have seen tremendous advances in *Urochloa* breeding, challenges in understanding the taxonomy and evolutionary history in *Urochloa* species remain and limit breeders to a relatively small pool of taxa and accessions to choose from, which slows down novel breeding initiatives (do Valle *et al.*, 2013; Sweitzer *et al.*, 2021). As one of the most economically significant forage genera across the tropics, improving the sustainability of *Urochloa* cultivars while maintaining their high productivity is paramount for achieving development goals.

*Urochloa* cultivar breeding is difficult due to asexual reproduction via apomixis and diverse ploidy levels present in most genotypes grown as forages (Miles and do Valle, 1996; Miles, 2007; Worthington and Miles, 2015; Worthington *et al.*, 2016; Hanley *et al.*, 2021; Tomaszewska *et al*., 2021). To overcome the challenges posed by apomixis and polyploidy, forage breeders artificially induce polyploidy in sexually reproducing diploids and cross them with closely related apomictic polyploid species (Ishigaki *et al.*, 2009). For example, tetraploids of the apomictic species *U. brizantha* and *U. decumbens* are crossed with artificial tetraploids of the sexual diploid *U. ruziziensis*, where apomicts act as the pollen donors (Miles and do Valle, 1996). Crosses of these three species have produced the most commercially successful *Urochloa* cultivars (Pizarro *et al.*, 2013). Exploiting interspecific hybridization is central to modern *Urochloa* forage breeding, and successful cultivar production requires in-depth knowledge of the taxonomy and ploidy of the species used for crossing. Molecular data have revealed the shared, complex evolutionary history of *U. brizantha*, *U. decumbens* and *U. ruziziensis*. While all three species are clearly closely related, *U. brizantha* and *U. ruziziensis* belong to divergent lineages while *U. decumbens* is probably paraphyletic and split between two lineages based on ploidy; that is, tetraploid *U. decumbens* populations are more closely related to *U. brizantha* and diploid *U. decumbens* populations are more closely related to *U. ruziziensis* (Triviño *et al.*, 2017; Higgins *et al.*, 2022; Tomaszewska *et al.*, 2023).

Taxonomic uncertainty, mosaics of sexually and asexually reproducing close relatives, and diverse intraspecies ploidy levels are typical of *Urochloa* forage species, and forage grasses more generally (Sandhu *et al*., 2015; Ortiz *et al*., 2020). This is the case for the largely apomictic, hexaploid species *U. humidicola*, the third most agriculturally significant *Urochloa* crop species after *U. brizantha* and *U. decumbens* (Vigna *et al*., 2016; Worthington *et al*., 2019). *Urochloa humidicola* populations include intermediates morphologically similar to *U. dictyoneura*, and taxonomists have argued that the two species be lumped (Sosef, 2016). Two additional agricultural species complexes (containing *U. mutica* with *U. arrecta*, and *U. trichopus* with *U. mosambicensis* respectively) also display overlapping morphologies, and apomictic reproduction (Toutain, 1986; Pereira Filho *et al.*, 2013). A further complication is that the modern taxonomic concept of *Urochloa*, a monophyletic clade comprising previously separate genera, now includes *Megathyrsus maximus* [synonyms *Panicum maximum* Jacq. and *U. maxima* (Jacq.) R.D. Webster], a globally significant tetraploid forage and weed species indigenous to the African continent (Salariato *et al*., 2012; Rhodes *et al*., 2021).

Although belonging to different species complexes, *Urochloa* forage species share numerous agriculturally relevant traits (Keller-Grein *et al*., 1996). These important forages display perennial growth habits, a trait directly associated with carbon sequestration (Lal, 2004; Wilson *et al*., 2018; Ledo *et al*., 2020). Generally, *Urochloa* forage grasses are characterized by their tall culms, large and broad leaves, and relatively large seeds (Fisher and Kerridge, 1996; Clayton *et al*., 2016). Grasses which naturally produce high yields for above-ground biomass are desirable in tropical forage systems, while the production of large and easily harvested seeds is an advantageous trait for seed multiplication (Juntasin *et al.*, 2022). *Urochloa* cultivars are predominantly sold and distributed through seed, and high yields in vegetative biomass and easy multiplication through seed are a dual aim for forage breeders (Hopkinson *et al*., 1996; Santos Filho, 1996; Ghimire *et al*., 2015). The inflorescences of forage species typically consist of simple, unbranched racemes (Reinheimer and Vegetti, 2008; Salariato *et al*., 2010), and with laterally elongated or ‘winged’ rachises (see Fig. 1A) (Clayton and Renvoize, 1982; Renvoize *et al*., 1996). These specific traits are not only agriculturally significant, but they are also directly measurable in wild *Urochloa* species through herbarium collections, or they are recorded in taxonomic treatments and floras (Clayton and Renvoize, 1982). Thus, modelling the evolution of these forage traits across *Urochloa sensu lato* (*s.l.*) is possible and can be used to identify wild species/clades with forage potential. To achieve this, a comprehensive and robust phylogeny for *Urochloa* is required.

Phylogenetic studies of *Urochloa* and all subsumed genera (hereafter *Urochloa s.l.*) have been limited to only a select few species, focusing largely on relationships between known economically important species, namely *U. brizantha*, *U. decumbens* and *U. humidicola* (Torres González and Morton, 2005; Pessoa-Filho *et al.*, 2017), or have sampled the genus broadly but with only a handful of chloroplast genes and/or nuclear barcoding markers for tree inference (Salariato *et al*., 2010; Washburn *et al*., 2015; Hackel *et al*., 2018). Understanding the evolution of important forage species in *Urochloa s.l.* requires a phylogeny inferred across a broad representation of the genus using a large set of independently evolving gene regions. Modelling the evolutionary history of *Urochloa* with such a dataset will allow us to accurately infer speciation events in the face of population dynamics such an incomplete lineage sorting (Maddison, 1997; Edwards, 2009) and account for gene duplications due to polyploidization events (Wendel, 2015).

*Urochloa* forage breeders have long recognized the urgent need to increase the pool of genetic diversity used to develop novel cultivars (do Valle *et al*., 2013; Alves *et al.*, 2014). In an effort to improve disease resistance, abiotic stress tolerance and sustainability of future crops, breeding projects now routinely look to cross crop plants with their nearest wild, non-domesticated relatives, rapidly bringing novel traits from wild populations into crops (Alves *et al.*, 2014; Beloni *et al.*, 2018). These closely related wild species have been termed ‘crop wild relatives’ (CWRs) (Harlan and De Wet, 1971), and their utilization in *Urochloa* forage breeding is underexploited. Potential CWR species have been identified in *Urochloa* based on inflorescence morphology (Renvoize *et al*., 1996), but to our knowledge these relationships have not been tested. A genus-wide phylogeny for *Urochloa* grasses would help confirm the placement of CWRs, and provide a starting point for expanding upon the species currently used in artificial hybridizations for *Urochloa* cultivar development.

We aim to infer a genus-wide phylogeny (species tree) for *Urochloa s.l.* using the Angiosperm 353 target enrichment of nuclear genes (Johnson *et al*., 2019) and infer the placement of the following agriculturally important species: *U. brizantha*, *U. decumbens*, *U. ruzizie*nsis, *U. humidicola*, *U. mutica*, *U. arrecta*, *U. trichopus*, *U. mosambicensis*, and *M. maximus*. We aim to define CWRs for all *Urochloa* forage species based on well-supported recent common ancestry and monophyly. Finally, we infer ancestral trait estimates along a phylogeny for the following agriculturally important traits: perennial vs annual life cycle, culm height, leaf size, rachis wing morphology and seed size. This will allow us to estimate the emergence of species with forage potential across *Urochloa s.l*.

## MATERIALS AND METHODS

### Taxon sampling

A total of 64 samples representing 60 species were chosen for phylogenomic analysis using the Angiosperm 353 target capture probe set (Johnson *et al*., 2019). The complete dataset contained 54 species within *Urochloa s.l.* (including samples from the genera *Brachiaria*, *Eriochloa*, *Megathyrsus* and *Scutachne*) and includes six *Brachiaria* species that have been placed within the subtribe Boivinellinae (Hackel *et al.*, 2018) in the tribe Paniceae. These species are *B. antsirabensis* A. Camus, *B. bemarivensis* A. Camus, *B. dimorpha* A. Camus, *B. epacridifolia* (Stapf) A. Camus, *Brachiaria* sp. (MSV_387) and *B. tsiafajavonensis* A. Camus. *Poecilostachys oplismenoides* (Hack.) Clayton was also included as a species within the Boivinellinae. To further test the generic limits of *Urochloa s.l.* we included five additional species within the subtribe Melinidinae namely *Melinis repens* (Willd.) Zizka, *Moorochloa eruciformis* (Sm.) Veldkamp, *Moorochloa malacodes* (Mez & K.Schum.) Veldkamp, *Tricholaena monachne* (Trin.) Stapf & C.E.Hubb. and *Thuarea perrieri* A. Camus. *Anthephora hermaphrodita* (L.) Kuntze was chosen as the outgroup taxon within the tribe Paniceae (see Kellogg, 2015 and; Soreng *et al*., 2017 for classification within Poaceae).

Of the 64 total samples, 11 were downloaded as raw RNA reads from the European Nucleotide Archive (ENA, https://www.ebi.ac.uk/ena/browser/home). Raw reads for a further 23 samples were obtained from Baker *et al.* (2022). The remaining samples were selected from herbarium and silica-dried material available from the Royal Botanic Gardens, Kew. Taxa were chosen to meet two objectives: sample species broadly across *Urochloa s.l.* and to target potential CWRs. The Kew Herbarium index, which orders species within genera based on morphological similarities, as well as Renvoize and Maass (1993) and Renvoize *et al.* (1996) were used as guides for potential CWR sampling. A summary of all samples used with metadata for downstream analysis and data sources can be found in Supplementary Data Table S1. Leaf material from herbarium and silica-dried specimens was then used for DNA extraction and target enrichment library preparation.

### Extraction and target-enrichment sequencing

#### DNA extraction, library preparation and sequencing.

DNA extraction was performed using a modified CTAB protocol (Doyle and Doyle, 1987). Quantus Fluorometer (Promega, Madison, WI, USA) measurements and gel electrophoresis using a 1 % agarose gel were conducted to estimate extraction quality and DNA fragment size. Samples with average fragment sizes estimated to be larger than 350 bp were sonicated using an M220 Focused ultrasonicator (Covaris, Woburn, MA, USA). Library preparation was then conducted following the NEBNext Ultra II DNA library prep kit protocol, performing half volume reactions, using Primer sets 1 and 2, and NEBNext Multiplex Oligos from Illumina. Following adaptor ligation and size selection, samples were PCR amplified for eight cycles. Library concentrations were assessed using a Quantus Fluorometer and average fragment lengths were measured with the Agilent Technologies 4200 TapeStation (Santa Clara, CA, USA) using High Sensitivity D1000 ScreenTape. Samples with low concentrations were re-amplified for —eight or nine PCR cycles. Libraries were then pooled depending on concentrations and fragment sizes, resulting in four pools of fragment lengths 240–269, 270–330, 340–395 and 400–500 bp. Pooled libraries were normalized for equimolarity and hybridized with the Angiosperm 353 probes (Johnson *et al.*, 2019) for 24 h at 65 °C followed by 12 cycles of PCR. Final products were then assessed for fragment length using the Agilent Technologies 4200 Tapestation with D1000 ScreenTape before being sent to Macrogen Inc. (Seoul, South Korea) for sequencing on an Illumina HiSeqX platform (2 × 150-bp paired-end reads).

### Phylogenomic inference

#### Read processing and loci assembly.

Paired-end and unpaired raw reads for newly sequenced samples and samples obtained from ENA were trimmed of adapters and filtered for low quality using Trimmomatic 0.39 (Bolger *et al.*, 2014). A Phred33 score was specified for all samples. Trimming was performed by assessing the leading and trailing read ends, and a sliding window of 4 bp was used across reads. Base pairs falling below the quality threshold of 30 were removed for leading, trailing and sliding window trimming (LEADING:30; TRAILING:30; SLIDINGWINDOW:4:30). A minimum read length for reads following trimming was set to 36 bp (MINLEN:36).

Trimmed reads were used for loci assembly using HybPiper 1.3.1 (Johnson *et al.*, 2016). An amino acid target fasta file from Baker *et al.* (2022) was used to capture on-target reads for Angiosperm 353 loci using BLASTX v.2.5.0 (Camacho *et al.*, 2009). This was to ensure synonymous mutations did not bias read mapping to the target files across divergent taxa. Loci were then assembled *de novo* using SPAdes (Bankevich *et al.*, 2012) and retrieved for each sample using the ‘reads_first.py’ and ‘retrieve_sequences.py’ scripts from HybPiper. Only the exons of assembled genes were used for downstream phylogenomic analysis. This was chosen to standardize alignments in the dataset which contained both target-enrichment and transcriptomic sequences.

#### Paralogue removal and consensus sequence inference.

In target-enrichment-based phylogenomic inference, paralogous genes are commonly removed from the analysis as they introduce homoplasy and confound estimations of species divergence histories (Nicholls *et al*., 2015; Andermann *et al*., 2020; Larridon *et al*., 2020; Crowl *et al.*, 2022). Further, the presence of paralogues and duplicated genes can confound gene assembly, as reads from different paralogues can be adjoined into contigs (and subsequently genes), leading to chimeric sequence assembly (Kates *et al*., 2018; Nauheimer *et al*., 2021). However, removing entire genes with suspected paralogy means that genes with high allelic diversity (i.e. in the case of whole genome duplication and reticulation events) can be purged, resulting in the loss of informative gene sequences (Morales-Briones *et al*., 2022). Whole genome duplications and interspecific reticulation events are ubiquitous in angiosperms, and specifically prevalent in Poaceae and *Urochloa* (Van De Peer *et al*., 2009; McKain *et al*., 2016; Vigna *et al*., 2016; Landis *et al*., 2018).

To ensure that highly paralogous genes were removed while maintaining a large gene dataset, we assessed the heterozygosity of the 353 loci in our dataset based on the distribution of single nucleotide polymorphisms (SNPs) across all genes using HybPhaser (Nauheimer *et al*., 2021). On-target reads were mapped to loci recovered from HybPiper using BWA v.0.7.17 (Li and Durbin, 2009). Then bcftools v.1.9 (https://samtools.githu*b.*io/bcftools/bcftools.html) was used to call SNP variants using the HybPhaser script ‘1_generate_consensus_sequences.sh’ with default parameter settings for minimum allele frequency, minimum coverage and minimum allele count. Across all samples, loci with an SNP diversity that was 1.5 times greater than the third interquartile range for the entire gene dataset were removed using the ‘1a_snp_count.R’ and ‘1b_assessment.R’ scripts. This method allowed us to assess relative heterozygosity across *Urochloa* loci and flag outlier genes as potential paralogous, while retaining genes with multiple copies as a result of polyploidization events.

Once putative paralogous genes were removed, we phased the remaining gene set in order to infer non-chimeric consensus sequences following a method outlined in Tiley *et al.* (2021, 2023) and Crowl *et al.* (2022). Ploidy levels for all samples were required for the pipeline, which were obtained from the literature (Morrone *et al*., 2006; Tomaszewska *et al*., 2021) and the Chromosome Count Database (http://ccdb.tau.ac.il/), or estimated from target-enrichment sequencing reads (Tiley *et al.*, 2021, 2023). Ploidy levels were estimated by mapping reads to a reference sequence, in this case loci from the known diploid *U. fusca* (Morrone *et al*., 2006). Samples are initially genotyped as diploids using GATK v.3.8.1 (McKenna *et al*., 2010) and biallelic reads were then mapped to *U. fusca* loci. The ratio between reads matching the reference sequence and reads carrying the alternate were used to estimate ploidy levels (Tiley *et al.*, 2018). For ploidy estimation, a maximum ploidy level of 6 was chosen to constrain the analysis.

Following ploidy estimation, samples were phased for gene variants with the maximum number of possible haplotypes determined by the estimated ploidy levels. For each sample raw reads were assigned to respective gene sequences from HybPiper using BWA 0.7.17 (Li and Durbin, 2009) and PCR duplicates were flagged using Picard v.2.27.4 (http://broadinstitute.github.io/picard). HaplotypeCaller in GATK3.8.1 was used to assign reads to gene variants with parameter settings left to default (Tiley *et al*., 2021, 2023; Crowl *et al.*, 2022). Phased gene variants were then assembled using H-PoPG v.0.2.0 (Xie *et al.*, 2016). Phased variants were then collapsed into new consensus sequences where polymorphic sites were coded with ‘N’ to ensure that chimeric assemblies of homoeologous sequences were not present in phylogenetic analysis.

#### Species tree inference.

Newly inferred consensus sequences were aligned using MAFFT v.7.475 (Katoh and Standley, 2013) with parameters set to L-INS-I (--localpair; --maxiterate 1000) for the highest stringency. Columns in alignments with more than 30 % missing data were removed using Phyutility v.2.2.6. (Smith and Dunn, 2008). Individual maximum-likelihood genes (ML) trees were inferred using IQTREE v.2.1.2 (Minh *et al.*, 2020) with ModelFinder Plus (Kalyaanamoorthy *et al*., 2017) used to determine the best fit model per gene based on Bayesian Information Criterion (BIC) scores. Ultrafast bootstrapping was implemented to assess branch support using 1000 replicates (Hoang *et al.*, 2018). Gene trees were then concatenated into a single file and nodes with support values of ≤10 were collapsed using Newick Utilities v.1.6 (Junier and Zdobnov, 2010). Outlier taxa with excessively long branch lengths were then removed from each gene tree using TreeShrink v.1.3.9 (Mai and Mirarab, 2018) with the -b parameter kept at the default value of 5. Outlier taxa identified with TreeShrink were then removed from the original gene alignments. Gene trees were inferred from the new alignments using the same IQTREE parameters. A species tree was then inferred using ASTRAL-III v.5.7.7 (Zhang *et al*., 2018) with the unrooted gene trees used an input. Branch support was assessed using local posterior probabilities (LPP) and quartet scores (QS) using the -t2 flag in ASTRAL-III. Separately, gene alignments were concatenated into a supermatrix which was used to infer a ML phylogeny using IQTREE v.2.1.2. In total, 1000 ultrafast bootstrap replicates were used to assess branch support and a GTR+G+I nucleotide substitution model was chosen due to computational constraints.

### Character evolution

#### Trait data.

All species were scored for continuous and discrete character traits. Continuous traits assessed were maximum leaf area, maximum culm height and maximum seed size (inferred from maximum fertile lemma length). Discrete traits assessed were growth habit (annual vs perennial growth habit) and rachis wing morphology (wingless, narrowly winged or broadly winged). Trait data were obtained from GrassBase (Clayton *et al.*, 2016). Data were filtered for relevant species and traits using the tidyr (Wickham *et al*., 2023*a*) and dplyr (Wickham *et al*., 2023*b*) packages in R (R Core Team, 2023). Duplicate taxa and samples not identified to species level were also removed. Updating and reconciling species names between our samples and the GrassBase database was done using the World Checklist of Vascular Plants (WCVP) (Govaerts *et al*., 2021), accessed through Plants of the World Online (https://powo.science.kew.org/), and Vorontsova (2022).

#### Ancestral estimation methods.

Estimations for ancestral traits were inferred using the supermatrix ML tree. Taxa with no trait data and duplicate taxa were removed from the tree using the ‘drop.tip’ function in the R package ape (Paradis *et al*., 2004). For continuous traits (leaf area, culm height and fertile lemma length), values were log transformed and ML of ancestral states were estimated under a Brownian motion model using the fastANC function from the R package phytools (Revell, 2012). To account for uncertainty in trait estimations at nodes, the variance and confidence intervals for every node were also calculated (Losos, 1999). We then tested for phylogenetic signal, the tendency for closely related taxa to share trait values more frequently than by chance (Revell *et al*., 2008), in continuous traits using Blomberg’s *K* (Blomberg *et al*., 2003) and Pagel’s λ (Pagel, 1999).

To determine the best fit model for growth habit we compared ‘Equal Rates’, ‘All Rates Different’, ‘Perennial to Annual’ (but not reversible) and ‘Annual to Perennial’ (but not reversible) models following Revell and Harmon (2022). The model with the best Akaike Information Criterion (AIC) score was chosen for analysis. The same approach was used for rachis wing morphology. The models compared were ‘Equal Rates’, ‘All Rates Different’ and ‘Symmetrical Rates’, following Revell and Harmon (2022). Ancestral state estimations were then inferred using a marginal likelihood ancestral state reconstruction method using the R package corHMM (Beaulieu *et al.*, 2013). Posterior probabilities for ancestral states were then mapped as pie charts to internal nodes of the tree using the R package ggtree v.3.0.2 (Yu *et al*., 2017).

## RESULTS

### Sequence recovery, paralogue removal and consensus sequence inference with ploidy

Between 0.84 million and 16 million read pairs were sequenced in this study, with an average of 3.7 million read pairs. Angiosperm 353 gene recovery was high for all samples and ranged between 299 and 346 genes, or 84.7–98 %, and an average of 333 genes were recovered (94.3 %). HybPhaser was used to detect and remove highly paralogous (1.5 times higher than the third interquartile range for SNP percentages across all genes) genes which resulted in the removal of 20 putatively paralogous genes (Supplementary Data Fig. S1). Ploidy levels for all samples are given in Table S1 and the number of diploids and polyploids in the sample set is given in Fig. S2. The final gene alignment lengths ranged from 106 to 3502 bp with a mean alignment length of 791 bp. The final concatenated gene alignment had a sequence length of 257 747 bp.

### Phylogeny and origins of forages

**Fig. 2.**
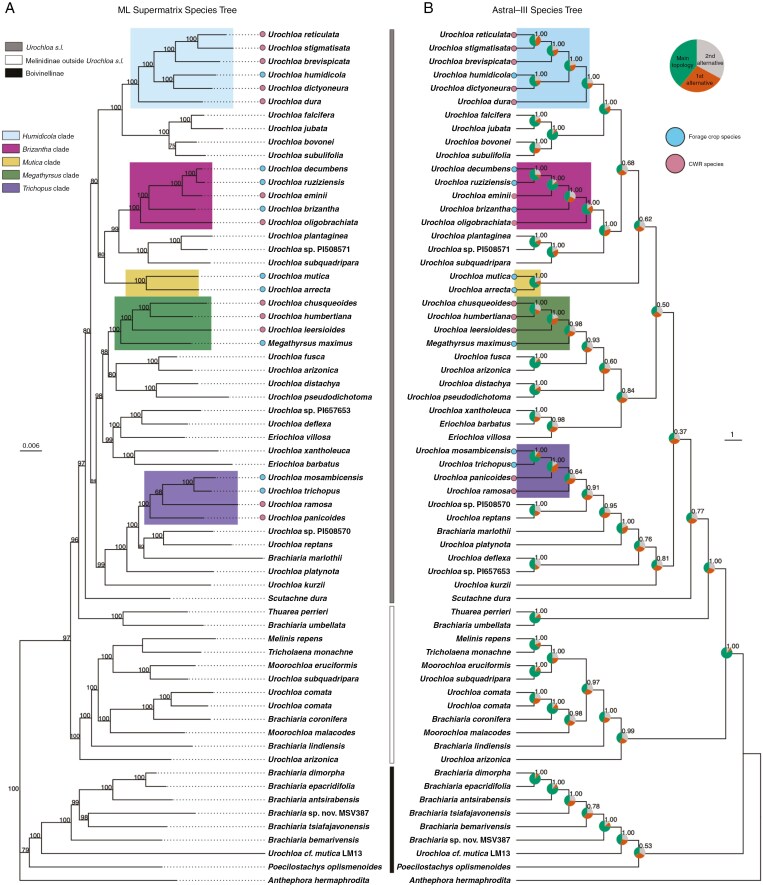
Phylogenetic inference of species trees for *Urochloa s.l.* using a maximum likelihood (ML) method on a concatenated alignment supermatrix using IQTREE2 in (A) and a multispecies coalescent method using ASTRAL-III (B). Numbers above branches in the ML phylogenetic tree (A) represent ultrafast bootstrap support values. For the ASTRAL-III phylogenetic tree (B) branch support is measured as local posterior probability (LPP) plotted above branches, and quartet scores (QS) were calculated and plotted on each node as pie charts. Colours in pie charts represent the main topology (green), the first alternative topology (orange) and the second alternative topology (grey). Forage clades are highlighted in colour in the ML phylogenetic tree (A) and ASTRAL-III phylogenetic tree (B). Scale bar for ML phylogenetic tree (A) indicates nucleotide substitutions per site, and for the ASTRAL-III phylogenetic tree (B) the scale bar indicate coalescent units. Forage species are indicated with blue dots at tips and CWR species are indicated at tips with pink dots. Taxa defined as *Urochloa s.l.* in the analysis are indicated with a dark grey bar. Taxa defined as sitting outside *Urochloa s.l.* but within the same subtibe Melinidinae are indicated with a white bar. Taxa within the Boivinellinae, including *Brachiaria* species endemic to Madagascar previously placed in Boivinellinae (Hackel *et al.*, 2018), are indicated with a black bar.

Phylogenetic trees produced using ASTRAL-III and IQTREE (hereafter ASTRAL-III and ML phylogenetic trees respectively) have largely similar topologies, and both phylogenetic trees recovered identical clades containing forage species (Fig. 2). We used the congruence between the ASTRAL-III and the ML phylogenetic trees in recovering these clades to define forage species clades and their CWRs (see Fig. 2, Table 1 for clades and support values). *Urochloa humidicola* and *U. dictyoneura* form a clade with the wild species *U. brevispicata*, *U. stigmatisata*, *U. reticulata* and *U. dura*, hereafter referred to as the ‘*Humidicola* clade’. The clade is well supported with 100 % bootstrap support (BS) in the ML phylogenetic tree, and 1.00 LPP and moderate gene tree congruence of 54.6 (QS for the main topology) in the ASTRAL-III phylogenetic tree. The three most commercially important *Urochloa* forages, *U. brizantha*, *U. decumbens* and *U. ruziziensis*, formed a clade with wild species *U. eminii* and *U. oligobrachiata* with 100 % BS in the ML phylogenetic tree and 1.00 LPP in the ASTRAL-III phylogeny, forming the ‘*Brizantha* clade’. The QS score for the *Brizantha* clade was moderate at 64.8 support for the main topology.

**Table 1.** *Urochloa* forage crop clades and their crop wild relatives (CWRs) defined here. Comparisons among forage traits between forage species and CWR are summarized.

	Forage species	CWR species	Forage species traits	CWR native to African continent	CWR forage traits
*Brizantha* Clade	*U. brizantha* *U. decumbens* *U. ruziziensis*	*U. eminii* *U. oligobrachiata*	Perennial growth habit, tall culms, large leaves, broad or narrow winged rachis, large seeds	Yes	Annual growth habits, tall culms, large leaves, broad winged rachis, large seeds
*Humidicola* Clade	*U. humidicola*	*U. brevispicata* *U. dictyoneura* *U. dura* *U. reticulata* *U. stigmatisata*	Perennial growth habit, tall culms, medium leaves, narrow winged rachis, large seeds	Yes	Annual and perennial growth habits, medium culms, small to medium leaves, narrow winged rachis, medium to large seeds
*Megathyrsus* Clade	*M. maximus*	*U. chusqueoides* *U. humbertiana* *U. leersiodes*	Perennial growth habit, tall culms, large leaves, wingless rachis, large seeds	Yes	Annual and perennial growth habits, short to medium culms, short leaves, wingless rachis, small to medium seeds
*Mutica* Clade	*U. arrecta* *U. mutica*	N/A	Perennial growth habit, tall culms, large leaves, broad winged rachis, large seeds	Yes	N/A
*Trichopus* Clade	*U. mosambicensis* *U. trichopus*	*U. panicoides* *U. ramosa*	Perennial or annual growth habit, tall culms, large leaves, narrow winged rachis, large seeds	Yes	Annual growth habits, medium to large leaves, small seeds, medium to tall culms, wingless and narrow winged rachis

*Urochloa arrecta* and *U. mutica* form a well-supported clade in the ML phylogenetic tree with 100 % BS. The same topology was recovered in the ASTRAL-III phylogenetic tree with 1.00 LPP and 65 QS indicating moderate congruence among gene tree topologies. No CWR species were identified from our phylogenetic analyses, meaning *U. mutica* and *U. arrecta* are the only species placed in the ‘*Mutica*’ forage clade. The ASTRAL-III phylogenetic tree places the *Mutica* clade sister to the *Brizantha* clade and *Humidicola* clade, while the ML phylogenetic tree shows the *Mutica* and *Brizantha* clades are closely related and the *Humidicola* clade belongs to a sister lineage. However, branch support for these topologies is moderate in the ML phylogenetic tree with 80 % BS, and low in the ASTRAL-III phylogenetic tree with 0.63 LPP and 36.15 QS.

Both the ML and ASTRAL-III phylogenetic trees confirmed the placement of *M. maximus* within *Urochloa*, and *M. maximus* was placed within a clade containing CWR species *U. humbertiana*, *U. leersioides* and *U. chusqueoides* (*Megathyrsus* clade). The *Megathyrsus* clade is well supported (100 % BS in the ML phylogenetic tree and 0.98 LPP in the ASTRAL-III phylogenetic tree). However, ASTRAL-III phylogenetic analysis shows high gene tree incongruence for the clade with a QS of 40.2. *Urochloa trichopus* and *U. mosambicensis* formed a clade with CWR species *U. panicoides* and *U. ramosa.* This clade is defined as the *Trichopus* forage clade, and it is highly supported in the ML phylogenetic tree (100 % BS) but poorly supported in the ASTRAL-III phylogenetic tree (0.64 LPP) with a low QS value (37.8). Apart from three forage species within the *Brizantha* clade, all forage species belonged to independent lineages within the genus *Urochloa*.

In addition to the genera *Scutachne* and *Megathyrsus*, both phylogenetic trees (Fig. 2) support placing *Eriochloa* within *Urochloa* and place *Moorochloa* as a separate but polyphyletic genus. *Brachiaria umbellata* is closely related to the genus *Thuarea* (Hackel *et al.*, 2018) and sits outside the *Urochloa* clade in our analysis. In both phylogenetic trees (Fig. 2), our analysis supports the Boivinellinae clade, containing ‘*Brachiaria*’ species that are endemic to Madagascar, as sister to the subtribe Melinidinae (Hackel *et al*., 2018). Both trees show that *U. arizonica* and *U. subquadripara* are paraphyletic with an accession from each species falling within the *Urochloa* clade, and an additional accession for each species placed with the remaining Melinidinae taxa. A morphologically ambiguous accession which appears to be affiliated with *U. mutica* (Seteraneski s.n., barcode K001102413) is placed outside the *Urochloa* clade in the subtribe Boivinellinae. This accession is labelled ‘*Urochloa cf. mutica* LM13’ in Fig. 2.

### Forage trait ancestral state estimations

**Fig. 3.**
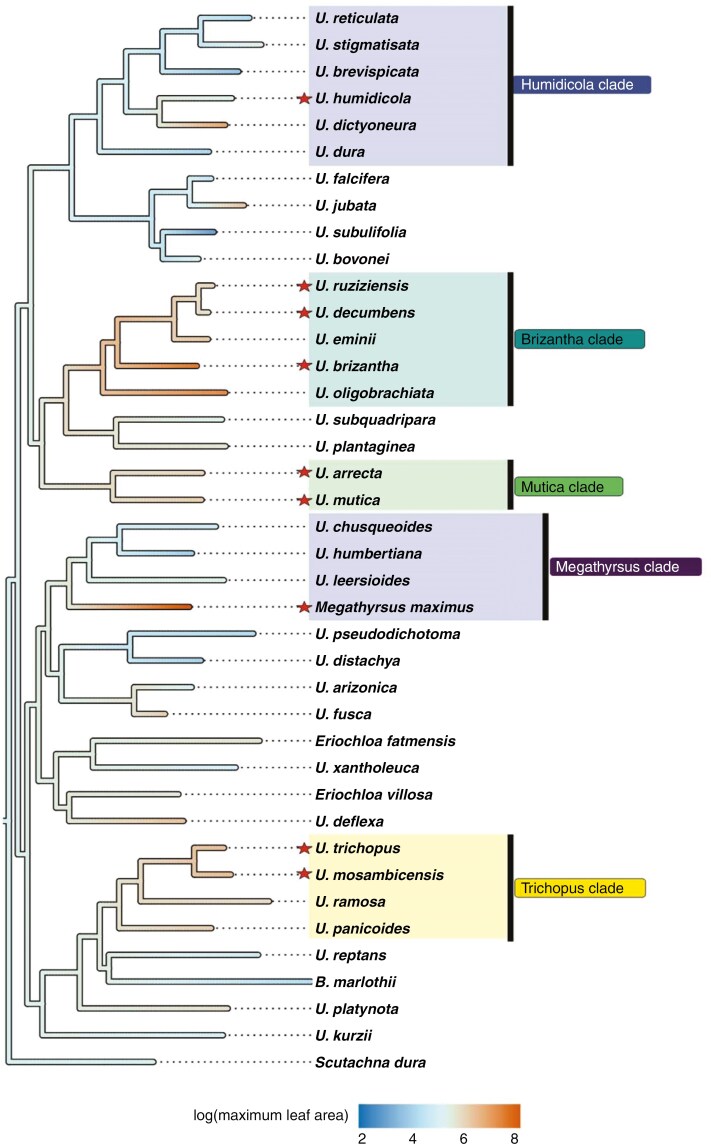
Evolution of log-transformed leaf area (cm^2^) in the *Urochloa s.l.* tree excluding Melinidinae, Boivinellinae and outgroup taxa. Forage species are marked with a star and forage clades are highlighted. Ancestral state estimations are inferred along branch lengths.

Ancestral states for the chosen continuous traits showed moderate size for leaf area, culm height and seeds (fertile lemma length) across not just *Urochloa*, but also the subtribe Melinidinae and Malagasy ‘*Brachiaria*’ species. For the *Brizantha* and *Mutica* clades, ancestral state estimations for log leaf area (Fig. 3, and Supplementary Data Fig S3) and log culm height ( Fig. S4) show an increase in size of these traits at the node of their respective most recent ancestors. The *Humidicola*, *Megathyrsus* and *Trichopus* clades show greater variability in these two traits, as agriculturally significant species differ from their closest relatives and their respective estimated ancestors. This is most observably clear for *M. maximus*, which evolved much larger leaves and culms than its closest living relatives and their most recent shared ancestor.

Estimates for log fertile lemma length (Supplementary Data Fig. S5) show *M. maximus*, *U. trichopus*, *U. mosambicensis* and *U. arrecta* evolved large seeds from small-seeded ancestors independently. The ancestral state for the *Humidicola* clade indicates that the common ancestor probably had moderately large seeds, though seed size increased along the *U. humidicola*/*U. dictyoneura* lineage and decreased in wild relatives. Long fertile lemma length estimates for the *Brizantha* clade indicate that this clade evolved from a common ancestor with large seeds. Phylogenetic signal for all three continuous traits was high and statistically significant, indicating that the similarity in trait values across all taxa is the result of a shared phylogenetic history (Table 2).

**Table 2.** Phylogenetic signal values (Blomberg’s *K* and Pagel’s λ) with *P*-values for natural log values of continuous traits (leaf area, lemma length and culm height).

	Blomberg’s *K*	*P*-value	Pagel’s λ	*P*-value
log Leaf area	0.775 067	0.0016	0.800 593	9.51344e^−5^
log Lemma length	0.68 713	0.0052	0.999 934	0.000952218
log Culm height	0.636 892	0.0254	0.891 587	0.00495678

**Fig. 4.**
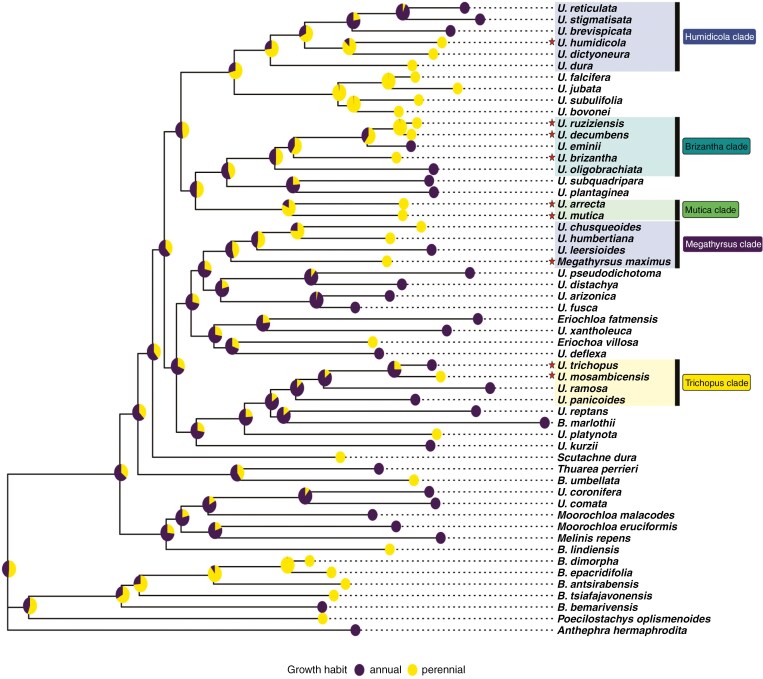
Evolution of growth form (annual vs perennial). Forage species are marked with a star and forage clades are highlighted. Ancestral state estimations for annual versus perennial habits were performed using corHMM in R and posterior probabilities for state estimations were mapped to ancestral nodes.

**Fig. 5.**
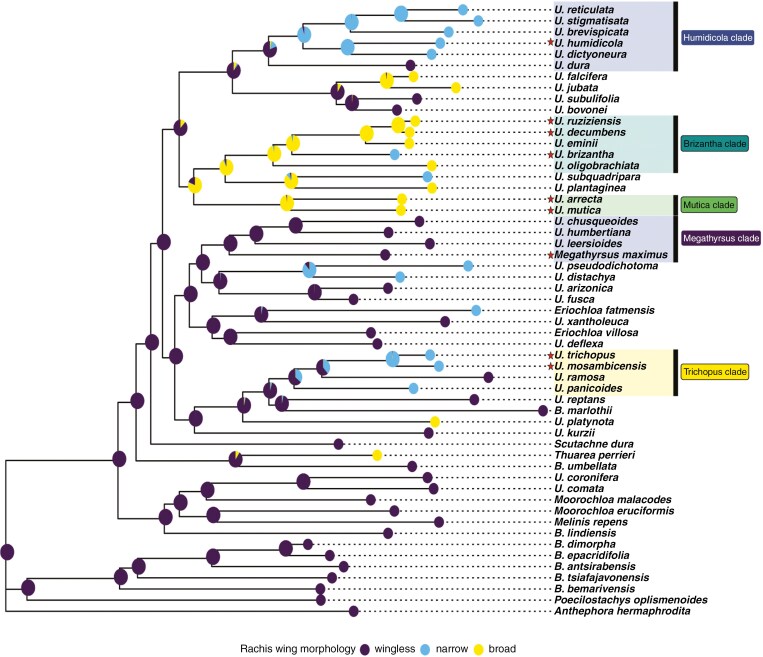
Evolution of rachis morphology (wingless, narrowly winged or broadly winged). Forage species are marked with a star and forage clades are highlighted. Estimates indicate that the common ancestor of extant *Urochloa s.l.* species did not have a winged rachis (purple), and that the widening of the rachis occurred multiple times within forage clades (blue and yellow for narrow and broad wings respectively). Ancestral state estimations were performed using corHMM in R and posterior probabilities for state estimations were mapped to ancestral nodes.

AIC scores determined that an ‘Equal Traits’ model was the best fit for ancestral trait estimation for both discrete traits (i.e. growth habit and rachis wing morphology) (Table 3). Posterior probability scores show that the ancestor of *Urochloa* grasses was probably an annual and that perennial growth habits probably emerged multiple times at ancestral nodes within the genus (Fig. 4). Estimations of ancestral rachis wing morphology show strongly that *Urochloa s.l.* evolved from an ancestor with a wingless rachis (Fig. 5). The emergence of a rachis wing (narrow or broad) occurred in parallel across multiple nodes in the phylogeny and is generally associated with forage clades (a notable exception being the *Megathyrsus* clade where all members are wingless).

**Table 3.** Model selection for growth habit (annual vs perennial) and rachis wing morphology with log likelihood, Akaike Information Criterion (AIC), and delta AIC scores.

Trait	Model	log likelihood	AIC	delta AIC
Growth habit	Equal Rates	−43.0951	88.19	0
	All Rates Different	−43.0951	90.19	2
	Annual to Perennial	−46.12 271	94.245	6.055209
	Perennial to Annual	−49.5135	101.03	12.8368
Rachis morphology	Equal Rates	−51.12 791	104.26	0
	All Rates Different	−47.87 176	119.74	15.4877
	Symmetrical Rates	−48.81 644	109.63	5.377 079

As the *Brizantha* and *Mutica* clades are the most closely related forage clades, posterior probabilities indicate that the shared ancestor of these important forage clades may have already evolved a broad rachis wing. For the *Humidicola* clade, results indicate that the clade probably evolved from a wingless ancestor, but that a narrowly winged form probably emerged at an early point in the clade’s divergence, as the vast majority of *Humidicola* clade species possess winged rachises.

## DISCUSSION

### Crop wild relatives for Urochloa forage breeding

The phylogenetic analyses conducted here provide a platform for interpreting the evolution of *Urochloa s.l*. A broad definition of *Urochloa s.l.* (which includes all subsumed genera) is supported in our ASTRAL-III species tree and ML supermatrix tree (Fig. 2), confirming previous results based on chloroplast markers (Salariato *et al.*, 2010, 2012; Hackel *et al.*, 2018; Delfini *et al.*, 2023). Further, both ASTRAL-III and ML trees recovered clades supporting infrageneric groupings in *Urochloa* based on morphological characters (Renvoize *et al.*, 1996). However, inferring trees using hundreds of nuclear loci allowed us to resolve polytomies previously recovered in *Urochloa s.l.* phylogenies inferred from chloroplast markers. For example, Salariato *et al*. (2010, 2012) and Delfini *et al.* (2023) consistently fail to recover the *Humidicola* and *Mutica* clades using chloroplast markers. The recovery of forage clades that reliably agree with morphological subgroups within *Urochloa s.l.* suggests our phylogenies provide more realistic species relations in the genus compared to previous studies.

For all forage species, we were able to identify CWRs based on monophyletic clades recovered in both ASTRAL-III and ML supermatrix analyses. CWRs are important taxa for agricultural purposes as they provide the comparative context for in-depth analysis for phenotypic developmental and molecular studies in crops (Harlan and DeWet, 1971; Pironon *et al*., 2020; Viruel *et al*., 2021). Additionally, crosses between crop species and their wild relatives have produced hybrid progeny with highly desirable agricultural traits, such as fungal and nematode disease resistance in peanuts (Bertioli *et al*., 2021), or increase heat and drought tolerance in wheat (Molero *et al*., 2023). Interspecific crossing is routine in *Urochloa* cultivar production as the most popular commercially available lines, Mulato and Mulato II, were developed from *U. brizantha* × *U. decumbens* × *U. ruziziensis* hybrids (Argel *et al.*, 2007; Pizarro *et al*., 2013).

*Urochloa brizantha*, *U. decumbens* and *U. ruziziensis* share a most recent common ancestor with CWR species *U. eminii* and *U. oligobrachiata*, confirming the observations of Renvoize *et al.* (1996) based on morphological data. Further, alpha taxonomists have long argued that intermediate specimens are common between *U. ruziziensis* and *U. decumbens*, and *U. decumbens* and *U. brizantha* respectively (Renvoize and Maass, 1993; Renvoize *et al.,* 1996; Sosef, 2016), demonstrating that detailed morphological studies still provide insight into evolutionary dynamics present in even highly reticulate species complexes. Sosef (2016) argued that *U. decumbens* and *U. ruziziensis* be lumped together along with *U. eminii*, and our analysis confirms that the three taxa are the most closely related species within the *Brizantha* complex. To our knowledge, *U. eminii* and *U. oligobrachiata* are not used in forage grass breeding programmes, but it is likely that these CWRs can be hybridized with *U. brizantha*, *U. decumbens* and *U. ruziziensis* to produce cultivars with novel phenotypes (though this will need to be tested empirically).

Cultivar development for *U. humidicola* forages lags behind the *Brizantha* clade at a commercial scale (Boldrini *et al.*, 2011). While apomictic reproduction in the complex has been overcome by the chance discovery of sexually reproductive accessions (Jungmann *et al.*, 2009), cultivars are developed by crossing sexual and apomictic *U. humidicola* accessions only (Jungmann *et al*., 2009; de Figueiredo *et al*., 2019; Berchembrock *et al*., 2020). High ploidy levels (hexaploidy and heptaploidy typically) in *U. dictyoneura* and *U. humidicola* and reduced genetic diversity in seed bank collections creates a stumbling block for breeders (Miles and do Valle, 1996; Vigna *et al.*, 2016; Higgins *et al.*, 2022). Introducing CWRs into breeding systems remains a viable option for overcoming these limitations, particularly for increasing genetic diversity. Renvoize *et al*. (1996) identified *U. stigmatisata*, *U. reticulata* and *U. brevispicata* as close relatives of *U. humidicola* and *U. dictyoneura*, which is supported by our phylogenetic analyses (Fig. 2). However, Renvoize *et al*. (1996) grouped *U. dura* with *Brizantha* clade species, whereas our analyses show that *U. dura* belongs to the *Humidicola* clade. Further, Renvoize *et al.* (1996) placed *U. platynota* as a close relative to the *Humidicola* clade, but our analysis shows it is a distantly related species. Finally, Renvoize *et al.* (1996) grouped *U. falcifera*, *U. jubata*, *U. subulifolia* and *U. bovonei* with *Humidicola* clade species in their analysis. Our results show that these taxa form a well-supported clade sister to the *Humidicola* clade (Fig. 2).

Broadly, a suite of CWRs and sister taxa to the *U. humidicola/U. dictyoneura* complex have been inferred in the literature and are strongly supported by our phylogenetic results (Fig. 2). Introducing CWRs and interspecific breeding into *U. humidicola*/*U. dictyoneura* cultivars could result in new forage lines with novel agricultural traits, as has already successfully been demonstrated in hybrid *Brizantha* clade cultivars (Argel *et al.*, 2007; Pizarro *et al.*, 2013). Additionally, cytological results and fluorescence *in situ* hybridization have provided evidence for the inferred allopolyploid origins of *U. humidicola* and, crucially, potential subgenome identification in the species (Vigna *et al*., 2016; Tomaszewska *et al.*, 2023). CWRs provide a sensible starting point for investigating the putative donors of *U. humidicola* subgenomes, as has been demonstrated across numerous allopolyploid crop species, such as bread wheat, strawberries and *Brassica* crops (He *et al.*, 2017; Edger *et al.*, 2019; Yim *et al.*, 2022).

*Trichopus* and *Mutica* clade species are less commercially important in a global context, though their importance as livestock feed at small scales has been noted (Fischer and Kerridge, 1996; Pereira Filho *et al*., 2013). *Urochloa trichopus* and *U. mosambicensis* have been shown to be a nutrient-dense food source for goats in low-precipitation regions of Brazil such as the Caatinga (do Santos Pessoa *et al*., 2022). While the *Trichopus* clade is distantly related to other forage clades, the *Mutica* clade shares a recent common ancestor with the *Brizantha* and *Humidicola* clade. The placement of *Megathyrsus* within *Urochloa* is strongly supported, and its placement with species with more broadly spaced and pedicelled spikelets (i.e. *U. chusqueoides* and *U. humbertiana*) (Renvoize *et al*., 1996) provides a sensible framework for comparative analysis of inflorescence diversity in *Urochloa s.l.* See Supplementary Data Table S2 for a summary of *Urochloa* CWR species and their traits.

### Agricultural trait evolution

Modelling character evolution is challenging in groups where data availability is limited for both taxa and appropriate traits. Herbarium accessions play a pivotal role in bolstering taxon representation in phylogenies, especially for groups with geographical ranges spanning multiple countries and continents such as *Urochloa* (Besnard *et al.*, 2007; Baker *et al.*, 2021; Larson *et al.*, 2023). Comprehensive phylogenies are commonly utilized for evolutionary and ecological trait comparisons in numerous biological disciplines (Revell and Harmon, 2022), but the application of these methods in assessing agricultural potential across plant species (and clades) is underexplored. Forage grasses present a unique opportunity to apply phylogenetic comparative methods for agricultural purposes as traits of interest for breeders, taxonomists and ecologists share considerable overlap (leaf size, plant height, growth habit, etc.) and are probably present in floras and databases (Clayton and Renvoize, 1982; Clayton *et al*., 2016). For select *Urochloa* species, the domestication process is well within the initial phases (Dusi *et al*., 2010; Jank *et al.*, 2014).

The three continuous traits assessed in this study (leaf area, culm height and seed size) had moderate to high values for Blomberg’s *K* and Pagel’s λ, and all values were statistically significant (Table 2). Values approaching 1 for both Blomberg’s *K* and Pagel’s λ are interpreted as high phylogenetic signal for the trait in question (Revell *et al.*, 2008). This is evidence that shared values for the continuous traits assessed are due to shared ancestry in *Urochloa* grasses. Ancestral trait estimates for continuous traits generally show size increases along branches from ancestral clade nodes for important forage species and their close relatives. Our discrete character state estimations show a similar trend. A winged rachis morphology emerged independently in all forage clades except for the *Megathyrsus* clade where all species (including *M. maximus*) have wingless rachises and lax inflorescences. A winged rachis has been noted to impose greater rigidity on spikelet ordering (Renvoize *et al*., 1996), and is partly associated with a ‘homogenized’ inflorescence morphology as outlined by Salariato *et al.* (2010) and Reinheirmer and Vegetti (2008). Further investigation of how inflorescence structure influences seed retention (non-shattering phenotype) in *Urochloa* is essential, as non-shattering is among the first selected traits in plant domestication for grain production (Konishi *et al*., 2006; Yu *et al.*, 2020). Estimating ancestral growth habit shows more state uncertainty, particularly at deeper nodes in the phylogeny. However, there remains evidence that perennial growth habits have evolved multiple times across *Urochloa* in forage clades. Improving certainty for node state estimates can be achieved by more dense sampling of *Urochloa* species in future.

Palatable perennial grasses are common across the African continent (Ezenwa *et al*., 2006; Bond, 2008), though the independent emergence of species with forage potential across *Urochloa* is notable. The goal of forage grass breeding is to develop cultivars with unique phenotypes to suit specific geographical and climatic regions, while not sacrificing nutritional content and biomass production (do Valle *et al*., 2013; Nguku, 2015). Achieving this goal sustainably will require selecting material from genetically diverse accessions and taxa (Ferreira *et al*., 2021). Based on our results, the independent evolution of forage syndromes across African grasses implies a high amount of taxonomic and genetic diversity that forage breeders can draw from for future cultivar development.

A viable strategy for exploiting *Urochloa s.l.* diversity for agricultural gain is to introduce CWR species into forage cultivar breeding programmes. It is important to emphasize that CWR species are naturally endemic to Africa and are distributed across numerous African countries. For example, *U. eminii* (*Brizantha* clade CWR) has a range spanning west, east and central Africa (POWO, https://powo.science.kew.org/). Within-species variation for CWRs would be highly informative to breeders, but little is known about the CWR species identified in this study. To best utilize the agricultural potential of *Urochloa* CWRs, efforts must be made to understand their trait and genetic diversity in the wild. This implies that natural populations of *Urochloa* CWRs must be identified and, crucially, conserved. Here we highlight a clear overlap between agriculture and conservation interests: the genetic diversity in *Urochloa* forage species and their CWR exists in African savannah grasslands for breeders to utilize, and so the conservation of African grasslands is vital for the future of sustainable forage grass breeding both in Africa and across the tropics.

### Future considerations

Despite containing the world’s most important tropical forage grass species, *Urochloa s.l.* still contains an enormous amount of agricultural potential that has not been explored. Introducing CWRs into future breeding programmes is a stepping stone towards improving commercially available grasses. Interspecific *Urochloa* crosses are only successful if ploidy levels between parental species match and at least one parent is sexually reproductive. Addressing these knowledge gaps will require high-quality, chromosome-scale genome assemblies for important forage species and CWRs (Risso-Pascotto *et al*., 2005; Simeão *et al*., 2021). Within the forage clades identified in this study, only chromosome-scale genome assemblies exist for *U. ruziziensis* (Pessoa-Filho *et al*., 2019, available online but analysis unpublished; Worthington *et al.*, 2021). Additional high-quality genome assemblies will be valuable and can be used to determine ancestral genome origins in polyploids and chromosome rearrangements in the various species with distinct chromosome numbers, and to provide reference genomes for alignment of polymorphic markers from reduced-representation sequencing.

Genome assemblies could reveal the genetic pathways associated with *Urochloa* invasiveness into non-agricultural land, an unfortunate trend seen in African grasses globally (Visser *et al*., 2016). For example, *Mutica* clade species have been introduced from African countries to various parts of the world with the putative aim of improving pastures for livestock rearing (Williams and Baruch, 2000). While these species clearly have good forage characteristics, as demonstrated in this study and elsewhere (Fischer and Kerridge, 1996; Veldkamp, 1996), little scientifically informed breeding has been attempted in *U. mutica* and *U. arrecta*, and the two species are commonly classified as invasive weeds outside the African continent (Boyden *et al*., 2019). Even in the commercially important *Brizantha* clade, *U. decumbens* is an aggressive invasive in the Cerrado, a dry savannah region in Brazil (Pivello *et al*., 1999). This is probably a consequence of the species’ early introduction to South America as a forage grass prior to the establishment of genetic breeding programmes (Pivello *et al.*, 1999; Barbosa *et al*., 2008). There exists a substantive link between forage potential and aggressive invasiveness in African grasses, and genomic resources could help mitigate this undesirable attribute (Daehler and Carino, 1998; Williams and Baruch, 2000; Cook and Dias, 2006; Barbosa *et al*., 2008; Barbosa, 2016).

Beyond this study, there is still a need for more in-depth knowledge of the basic biology and diversity in *Urochloa s.l.*, and greater emphasis must be placed on conserving and collecting wild *Urochloa* grasses, particularly in African countries. While commercial cultivars are predominantly utilized at large scale in South America (Jank *et al*., 2014; Maass *et al.*, 2015), African nations have begun reintroducing cultivars in beef, dairy and push–pull pest control systems with notable successes (Mutimura, 2012; Khan *et al*., 2014; Clémence-Aggy *et al*., 2021). As the centre of *Urochloa s.l.* diversity, natural populations of African species probably contain the genes and traits needed to tailor new cultivars for the specific and varying needs of farmers, livestock and ecosystems across African nations. Conservation of African grasses is a global sustainability imperative as African grasslands form the basis of ancient habitats (Bond, 2016; Solofondranohatra *et al*., 2020; Buisson *et al*., 2022), perform natural carbon sequestration (Vågen *et al*., 2005; Dobson *et al*., 2022), and support the livelihoods of millions of people and animals (Bengtsson *et al*., 2019). African grassland conservation will safeguard the biodiversity needed to address issues of economic development and food security, and *Urochloa* is a genus of primary consideration in this regard.

## CONCLUSION

We have reconstructed a nuclear phylogeny for the grass genus *Urochloa s.l.* that is both comprehensively sampled and data rich, focusing on forages and their relatives. Our phylogenomic analysis allowed us to infer the placement of key agricultural species within the genus and identify their closest wild relatives. Additionally, we were able to estimate the ancestral state of numerous agriculturally important traits and demonstrate their convergent emergence in agriculturally important lineages. *Urochloa s.l.* is a highly morphologically diverse genus replete with polyploidization events and a natural distribution spanning the near entirety of the southern hemisphere. These attributes make *Urochloa* a prime example of how African grasses should serve as model systems for studying complicated evolutionary events, how a strong taxonomic and phylogenetic foundation can aid these studies, and how this knowledge can facilitate more sustainable agricultural practices in countries where it is most required.

## SUPPLEMENTARY DATA

Supplementary data are available at *Annals of Botany* online and consist of the following.

Table S1: metadata for all samples used in this study including estimated ploidy levels, trait data and accession data where available. Table S2: forage clades, CWR and forage traits obtained from GrassBase (Clayton *et al.*, 2016). Figure S1: histogram and boxplot of putative paralogues in the Angiosperm 353 locus sequences for samples used in this study. Figure S2: bar graph of ploidy levels (estimated or taken from the literature) for all samples used in this study. Figure S3: ancestral trait estimation for leaf area. Figure S4: ancestral trait estimation for culm height. Figure S5: ancestral trait estimation for fertile lemma length.

mcae022_suppl_Supplementary_Materials
