# Supplementary material for: Phylogenomic analysis reveals five independently evolved African forage grass clades in the genus Urochloa
Source: Ann Bot. 2024 Feb 14;133(5-6):725–42. doi: 10.1093/aob/mcae022 (PMC11082517; doi:10.1093/aob/mcae022)
Supplement: mcae022_suppl_Supplementary_Materials [file mcae022_suppl_supplementary_materials.zip › mcae022_suppl_Supplementary_Figures_S1.pptx]

## Slide 1
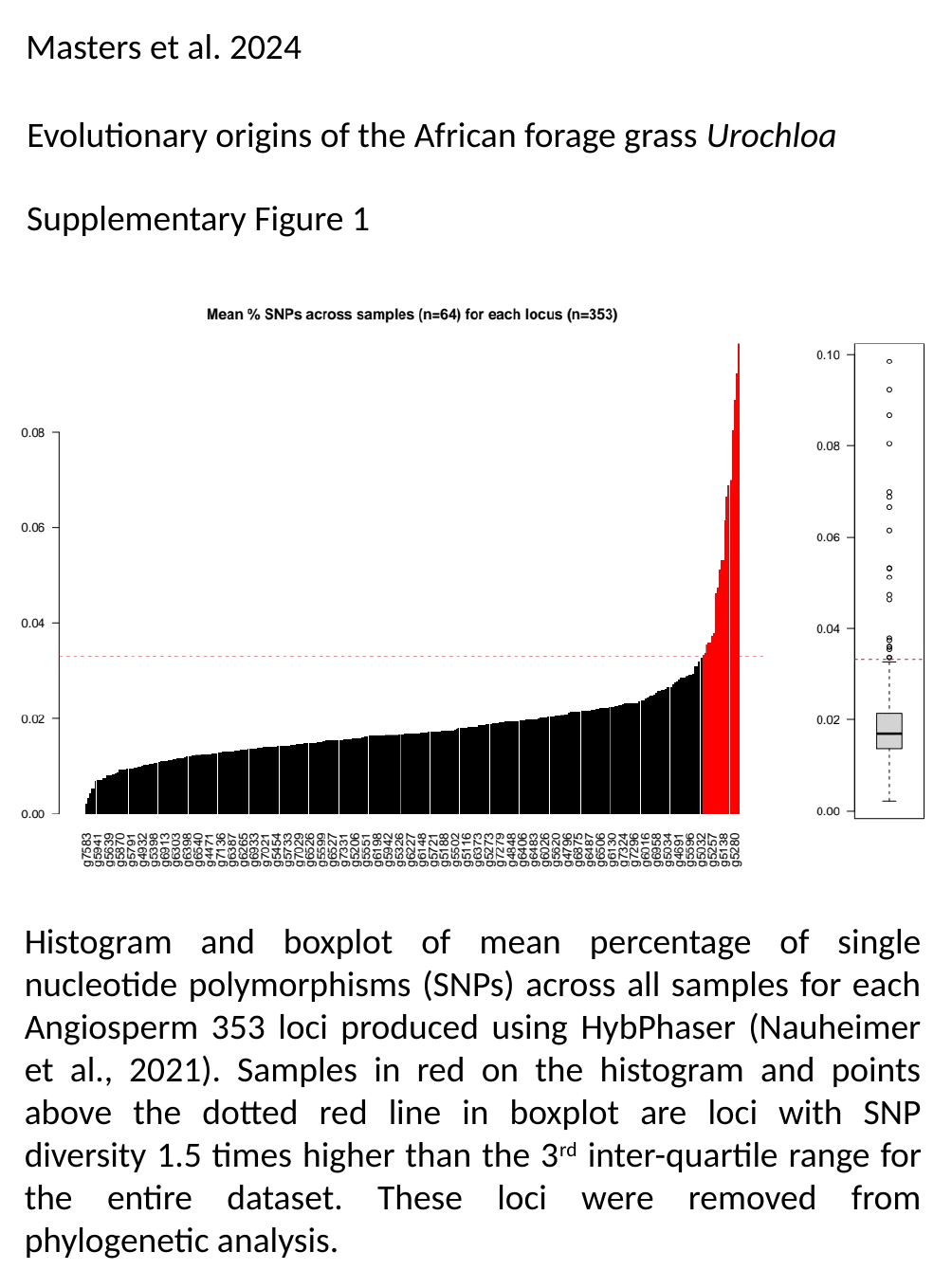

Masters et al. 2024
 Evolutionary origins of the African forage grass Urochloa
Supplementary Figure 1
Histogram and boxplot of mean percentage of single nucleotide polymorphisms (SNPs) across all samples for each Angiosperm 353 loci produced using HybPhaser (Nauheimer et al., 2021). Samples in red on the histogram and points above the dotted red line in boxplot are loci with SNP diversity 1.5 times higher than the 3rd inter-quartile range for the entire dataset. These loci were removed from phylogenetic analysis.
