# Supplementary material for: Phylogenomic analysis reveals five independently evolved African forage grass clades in the genus Urochloa
Source: Ann Bot. 2024 Feb 14;133(5-6):725–42. doi: 10.1093/aob/mcae022 (PMC11082517; doi:10.1093/aob/mcae022)
Supplement: mcae022_suppl_Supplementary_Materials [file mcae022_suppl_supplementary_materials.zip › mcae022_suppl_Supplementary_Figures_S2.pptx]

## Slide 1
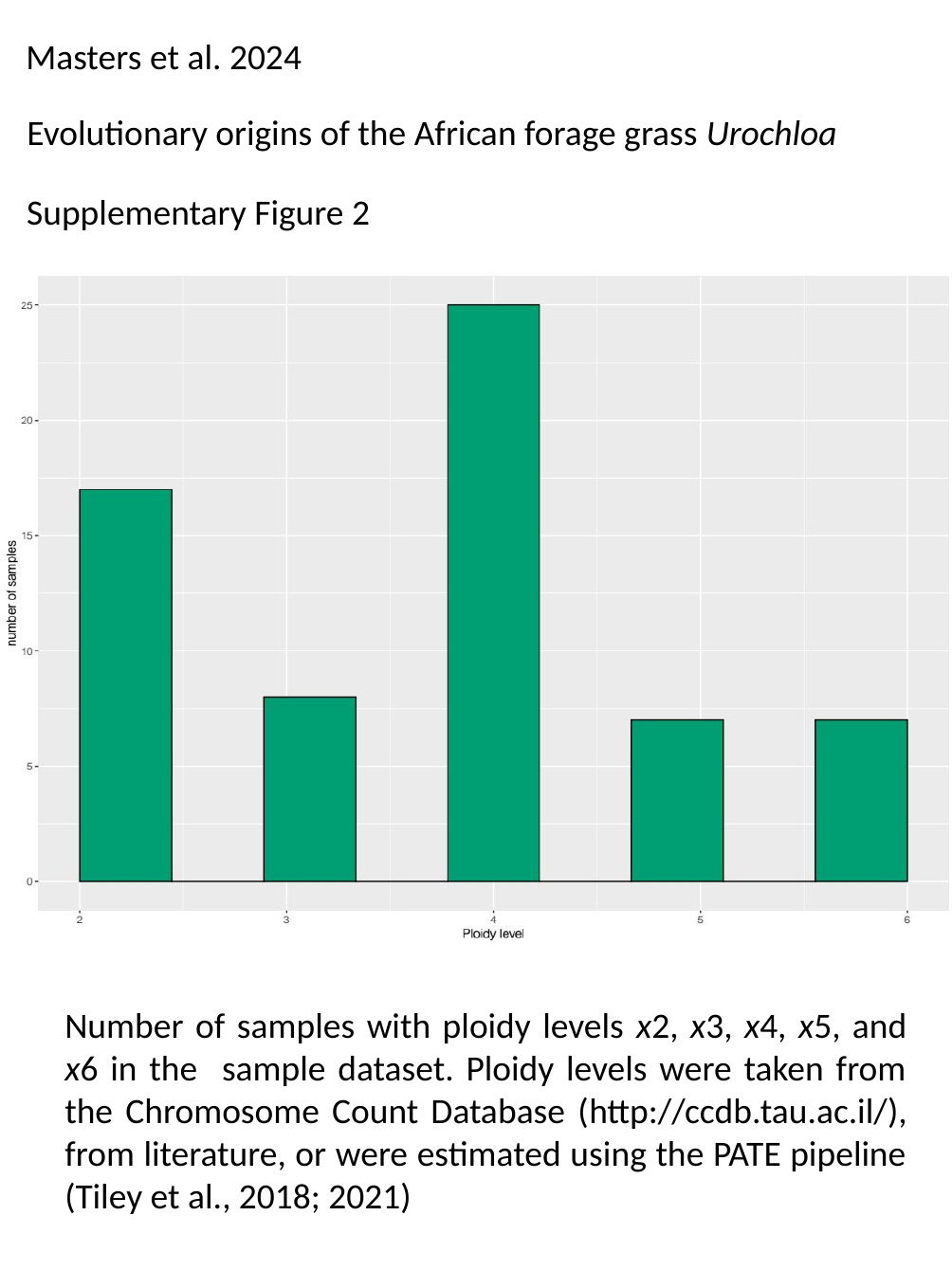

Masters et al. 2024
 Evolutionary origins of the African forage grass Urochloa
Supplementary Figure 2
Number of samples with ploidy levels x2, x3, x4, x5, and x6 in the sample dataset. Ploidy levels were taken from the Chromosome Count Database (http://ccdb.tau.ac.il/), from literature, or were estimated using the PATE pipeline (Tiley et al., 2018; 2021)
