# Supplementary material for: Phylogenomic analysis reveals five independently evolved African forage grass clades in the genus Urochloa
Source: Ann Bot. 2024 Feb 14;133(5-6):725–42. doi: 10.1093/aob/mcae022 (PMC11082517; doi:10.1093/aob/mcae022)
Supplement: mcae022_suppl_Supplementary_Materials [file mcae022_suppl_supplementary_materials.zip › mcae022_suppl_Supplementary_Figures_S3.pptx]

## Slide 1
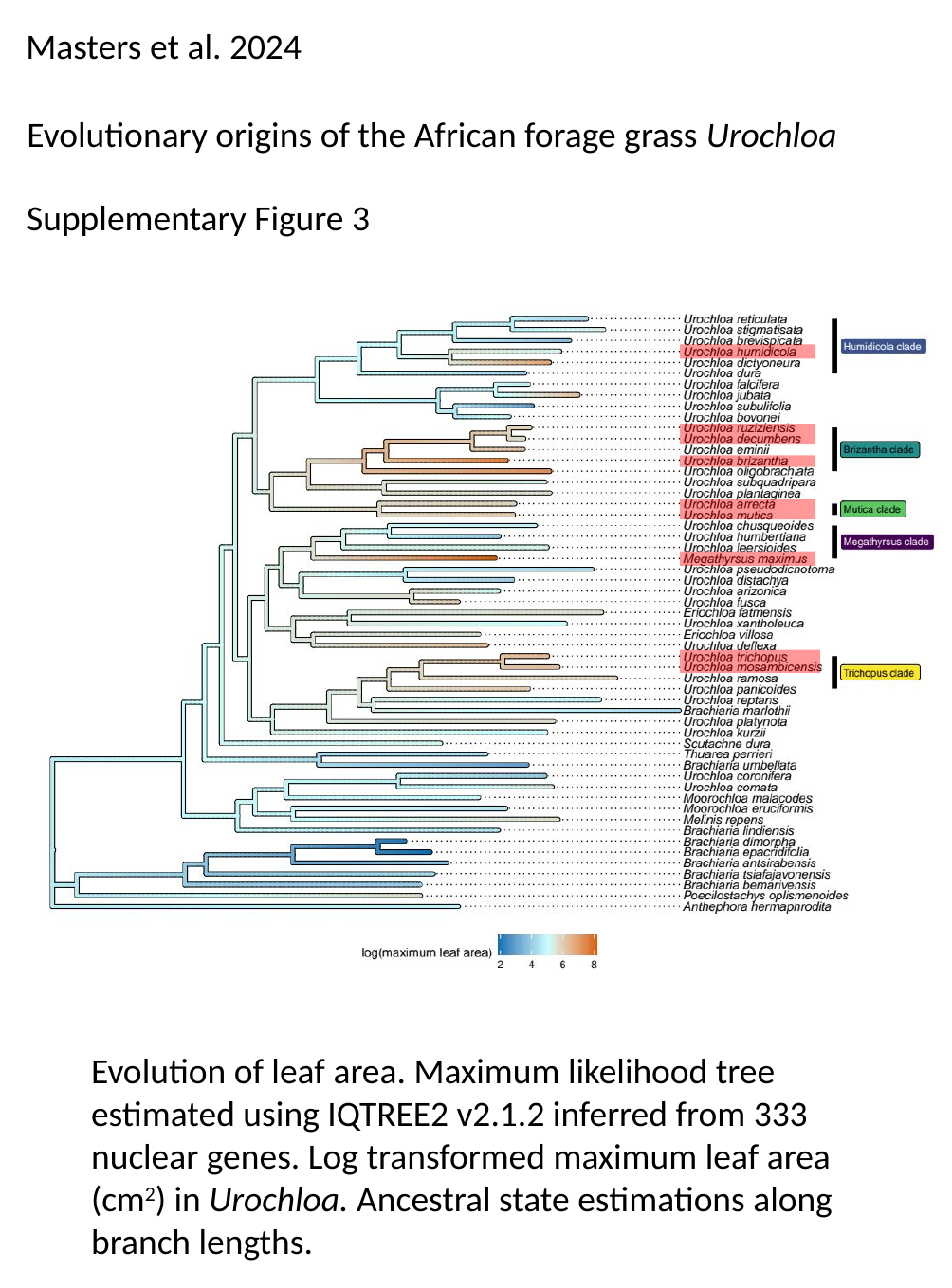

Masters et al. 2024
 Evolutionary origins of the African forage grass Urochloa
Supplementary Figure 3
Evolution of leaf area. Maximum likelihood tree estimated using IQTREE2 v2.1.2 inferred from 333 nuclear genes. Log transformed maximum leaf area (cm2) in Urochloa. Ancestral state estimations along branch lengths.
