# Supplementary material for: Phylogenomic analysis reveals five independently evolved African forage grass clades in the genus Urochloa
Source: Ann Bot. 2024 Feb 14;133(5-6):725–42. doi: 10.1093/aob/mcae022 (PMC11082517; doi:10.1093/aob/mcae022)
Supplement: mcae022_suppl_Supplementary_Materials [file mcae022_suppl_supplementary_materials.zip › mcae022_suppl_Supplementary_Figures_S5.pptx]

## Slide 1
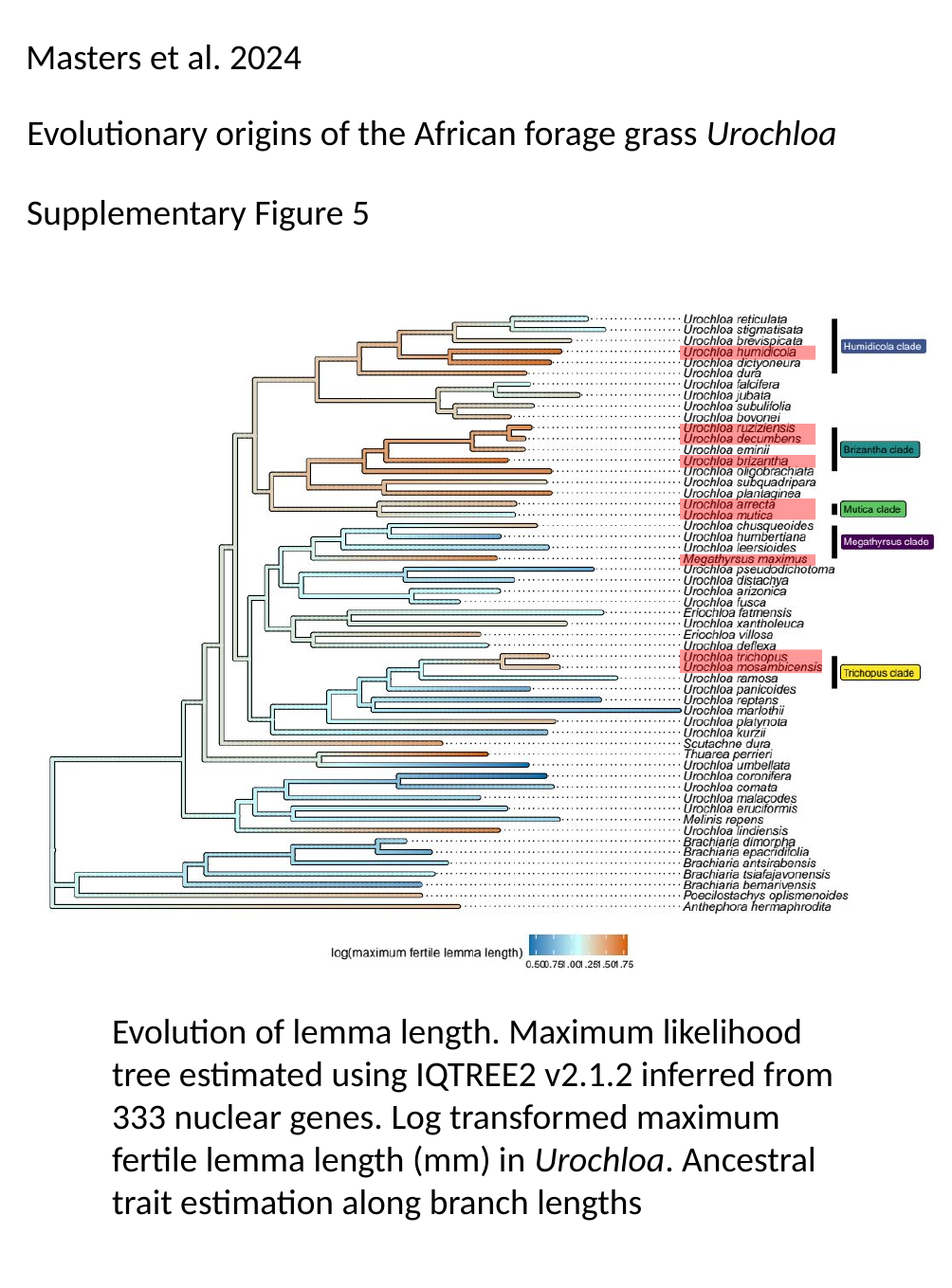

Masters et al. 2024
 Evolutionary origins of the African forage grass Urochloa
Supplementary Figure 5
Evolution of lemma length. Maximum likelihood tree estimated using IQTREE2 v2.1.2 inferred from 333 nuclear genes. Log transformed maximum fertile lemma length (mm) in Urochloa. Ancestral trait estimation along branch lengths
